# Modelling the effective dose to a population from fallout after a nuclear power plant accident—A scenario-based study with mitigating actions

**DOI:** 10.1371/journal.pone.0215081

**Published:** 2019-04-09

**Authors:** Mats Isaksson, Martin Tondel, Robert Wålinder, Christopher Rääf

**Affiliations:** 1 Department of Radiation Physics, Institute of Clinical Sciences, Sahlgrenska Academy, University of Gothenburg, Gothenburg, Sweden; 2 Occupational and Environmental Medicine, Department of Medical Sciences, University of Uppsala, Uppsala, Sweden; 3 Occupational and Environmental Medicine, Uppsala University Hospital, Uppsala, Sweden; 4 Medical Radiation Physics, Department of Translational Medicine, Malmö, Lund University, Malmö, Sweden; University of Kansas Medical Center, UNITED STATES

## Abstract

The radiological consequences of a nuclear power plant (NPP) accident, resulting in the release of radionuclides to the environment, will depend largely on the mitigating actions instigated shortly after the accident. It is therefore important to make predictions of the radiation dose to the affected population, from external as well as internal exposure, soon after an accident, despite the fact that data are scarce. The aim of this study was to develop a model for the prediction of the cumulative effective dose up to 84 years of age based on the ground deposition of ^137^Cs that is determined soon after fallout. The model accounts for different assumptions regarding external and internal dose contributions, and the model parameters in this study were chosen to reflect various mitigating actions. Furthermore, the relative importance of these parameters was determined by sensitivity analysis. To the best of our knowledge, this model is unique as it allows quantification of both the external and the internal effective dose using only a fallout map of ^137^Cs after a nuclear power plant accident. The cumulative effective dose over a period of 50 years following the accident per unit ^137^Cs deposited was found to range from 0.14 mSv/kBq m^-2^ to 1.5 mSv/kBq m^-2^, depending on the mitigating actions undertaken. According to the sensitivity analysis, the most important parameters governing the cumulative effective dose to various adult populations during 50 years after the fallout appear to be: the correlation factor between the local areal deposition of ^137^Cs and the maximum initial ambient dose rate; the maximum transfer from regional average fallout on the ground to body burden; the local areal deposition of ^137^Cs; and the regional average ^137^Cs deposition. Therefore, it is important that mapping of local ^137^Cs deposition is carried out immediately after fallout from a nuclear power plant accident, followed by calculations of radiation doses for different scenarios using well-known parameters, in order to identify the most efficient mitigation strategies. Given this ^137^Cs mapping, we believe our model is a valuable tool for long-term radiological assessment in the early phase after NPP accidents.

## Introduction

The consequences of the release of radionuclides to the environment following a nuclear power plant (NPP) accident will depend to a high degree on the steps taken to mitigate the effects shortly after the accident. Appropriate mitigating actions should thus be planned in advance by the relevant authorities in order to limit the consequences for both the individuals affected and society as a whole [1]. It is therefore important to make predictions of the radiation dose to the affected population over the long term, from both external and internal exposure, soon after an accident, even when few data are available. Estimates of the projected dose following various mitigating scenarios should therefore be made in advance; this was one of the main reasons for this study. The other was to develop a simple, yet comprehensive, relationship between the projected long-term radiation dose and a ground deposition parameter that can be determined relatively early after an accident by means of mobile measurements, e.g. airborne gamma surveys.

On 26 April 1986, an accident occurred at the NPP in Chernobyl, Ukraine. The total activity released during the first 10 days was estimated to be 5,300 PBq (excluding noble gases) [2]. In the ensuing days, 5% (4.25 PBq) of the ^137^Cs released was deposited in Sweden, especially during heavy rainfall on 28–29 April. Radionuclides were unevenly distributed in the eastern coastal regions from Stockholm in the south to Umeå in the north [3]. Hence, Sweden was the second most contaminated country, after the Soviet Union (areas now represented by the Russian Federation, Belarus and Ukraine). The ground deposition in the area of Sweden affected by the fallout exceeded 37 kBq ^137^Cs/m^2^ over an area of 12,000 km^2^ [2]. In our recently published study, by Jönsson *et al*., a model was presented describing the cumulative external effective dose from short-lived nuclides and the gamma-emitting Cs isotopes ^134^Cs and ^137^Cs in the most affected counties [4]. This model was developed using data from many databases: a) several time series of measurements of the ambient dose equivalent rate 1 m above the ground, to evaluate the time dependence of the total external exposure; b) air filter measurements from fixed continuous air gamma-rate monitoring stations; c) high-resolution gamma spectrometry data, to provide the initial nuclide composition of the fallout; and d) an aerial survey of the ground deposition of ^137^Cs in Sweden [5], to obtain the relationship between the initial ambient dose equivalent rate and the ground deposition of the long-lived gamma-emitting fission product ^137^Cs. According to this model, the predominant contribution to the external dose rate in the first month was from short-lived fission products, followed by ^134^Cs and subsequently ^137^Cs [4]. Integrated over a 70-year period, the model predicted that ^137^Cs would account for 60%, and ^134^Cs for 30%, of the projected external effective dose, and that the time-integrated external effective dose over 70 years, to an unshielded person resulting from all nuclides, per unit total activity of ^137^Cs deposition, would be 0.29 ± 0.08 mSv/(kBq per m^2^). The results obtained with this model were consistent with those given by similar models derived from retrospective dosimetry measurements in boreal forest areas in Russia [6], and we therefore hypothesized that this model can be generally applied in temperate climate zones. Although the model was based on an initial activity ratio between ^134^Cs and ^137^Cs in the Chernobyl fallout in 1986 of 0.56, replacing this value with the corresponding ratio from the Fukushima Daiichi NPP accident (1.1) showed that the 70-year integrated external dose would be only moderately affected (an increase of only 20%) [7]. Therefore, we believe that the previously developed model is also applicable for future releases, where the initial ^134^Cs/^137^Cs activity ratios will probably be more similar to that from the Fukushima accident.

Whole-body counting measurement programs have been carried out in Sweden since 1958, under the supervision of the Swedish Radiation Safety Authority and several Swedish universities (see e.g. [8]), to monitor various groups of the population. The average whole-body concentration of ^137^Cs for the reference urban population in the city of Stockholm was 1 Bq/kg in 1985. This value was used as a baseline when assessing the impact of the contribution from the Chernobyl NPP accident to the internal contamination of the studied groups by ^137^Cs. The body burdens soon increased, and peaked a year after the fallout, at 8 Bq/kg, returning to the pre-Chernobyl level of 1 Bq/kg in 2010. A database of average whole-body burdens in various population categories, such as urban residents, farmers, hunters and the Sami population, was constructed in 2006, and is available at the Swedish Radiation Safety Authority [9]. Using this database, Rääf *et al*. developed a model using transfer factors based on the deposition of ^137^Cs [10]. This model predicts a transfer factor for cumulative effective dose over a 70-year period resulting from internal contamination to the general (predominantly urban) population of 20–30 μSv/(kBq per m^2^). Hunters and farmers were identified as two groups with higher transfer factors, 100 μSv/(kBq per m^2^) and 50 μSv/(kBq per m^2^), respectively [10], reflecting various degrees of compliance with food intake recommendations and life style factors. When using these transfer factors, we found that the internal dose was about 75% of the total lifetime effective dose to hunters and their families [11]. The transfer factor for the urban population appears to be within a factor of two of that observed internationally [10]. However, as the consumption of game by Swedish hunters was relatively unregulated by the authorities, their exposure pathway and subsequent transfer factor is probably similar to the situation in which no mitigating actions were taken to reduce the ecological transfer of ^137^Cs to humans [11].

Given the observed agreement with international findings described above, we hypothesized that the model for calculating the lifetime cumulative internal and external effective dose (CED) to residents in affected areas could be applied in future radionuclide fallout situations, and was not restricted to the accident or the geographical area for which the model was initially developed. The aim of the present study was, therefore, to use the models for external and internal exposures mentioned above ([4], [10], [11]) to predict the long-term external and internal effective dose from fallout after any NPP accident for various scenarios including different mitigating actions, and to evaluate which parameters in the model have the greatest impact on the resulting cumulative effective dose.

## Materials and methods

### Description of the model

The effective dose resulting from external sources is directly dependent on the local areal activity concentration of ^137^Cs in the fallout, *A*_*esd*_, expressed as the equivalent surface deposition in the units kBq m^-2^ [12]. Equivalent surface deposition is defined as the areal activity density of a plane source that will lead to the same dose rate 1 m above the surface as the actual depth-distributed areal activity density, *A*_*tot*_ (kBq m^-2^). This definition is widely used in Sweden due to the ease of performing airborne measurements, rather than laborious soil sampling, for the assessment of the total activity deposition. The ratio between *A*_*esd*_ and the total Chernobyl ^137^Cs deposition density in Sweden, taking into account the fact that the soil inventory is underestimated when using the equivalent surface deposition, was found to be 1.6, based on measurements conducted by Edvarson et al. [13]. Hence, a relationship of *A*_*tot*_ = 1.6·*A*_*esd*_ can be applied in situations where ^137^Cs deposition mapping is mainly carried out by soil sampling, as was the case, for example, in Japan following the Fukushima Daiichi accident [14]. The initial ambient equivalent dose rate, ${\dot{H}}^{*}(t\;=\;0)$, resulting from fresh fallout, similar to the Chernobyl deposition in Sweden in terms of nuclide composition and ground penetration, has been modelled by Jönsson et al. [4], giving a value of the coefficient *d*_*Cs*_, the ambient equivalent dose rate per unit equivalent surface deposition, of 1.02±0.67 mSv y^-1^ per (kBq m^-2^). This value can be used to predict the initial external dose rates at various geographical locations, based on measurements of *A*_*esd*_ early after fallout, for example, by means of airborne surveys. If ^137^Cs deposition is mapped by soil sampling instead, the corresponding factor would be 1.6·1.02 = 1.63 mSv y^-1^ per (kBq m^-2^).

When modelling the time dependence of the external dose rate, the physical half-life, the migration properties and the resuspension of the various radionuclides in the fallout must be considered. The main contributors to the external dose rate in the longer term will be ^134^Cs and ^137^Cs, and the time dependence can then be modelled by the products *f*_*Cs-134*_*(t)·r(t)* and *f*_*Cs-137*_*(t)·r(t)*, respectively, where *f(t)* is the relative contribution from each Cs isotope, and *r(t)* is a normalized function describing the decrease in ambient dose equivalent rate with time. An exponential function, *r*(*t*), with four components was developed based specifically on the Swedish time series described by Jönsson et al. [4]. This function was derived from external dose rate measurements made every 7 months since 1986 at several locations in Sweden, continuous measurements of the gamma dose rate by 26 stationary detectors distributed throughout Sweden, and *in situ* measurements using high-resolution germanium spectrometers to determine the relative dose contributions from the radionuclides [4] and references therein. The normalized function describing the decrease in ambient dose equivalent rate with time *t* (years) is then given by:

$$r(t)\;=\;(0.96\pm 0.02)∙e^{-(37\pm 2)∙t}+(0.11\pm 0.01)∙e^{-(2.4\pm 0.6)∙t}+(0.080\pm 0.002)∙e^{-(0.67\pm 0.02)∙t}+(0.0314\pm 0.0008)∙e^{-(0.126\pm 0.002)∙t}$$ (1)

In contrast to the two-component function proposed by UNSCEAR [15], this four-component model allows for the combination of the time dynamics of the short-lived nuclides, as well as the difference in the physical half-lives of ^134^Cs and ^137^Cs when calculating the external dose contribution. The short-term dose rate will be determined mainly by short-lived gamma-emitting fission products, and can be described by the product [1- *f*_*Cs-134*_*(t)*-*f*_*Cs-137*_*(t)*]*· r(t)*. These functions are described in detail by Jönsson et al. [4]. The short-term contributions from cloud shine, inhalation and transfer of radioiodine through dairy milk have not been considered here, as these components are to a large extent avoidable, and are negligible for the long-term effective dose provided that early phase mitigating actions are carried out as recommended [1].

The relation between the ambient dose equivalent rate, ${\dot{H}}^{*}(t)$, and the air kerma rate, $\dot{K}(t)$, is given by the conversion coefficient h*_Emax_ [16], and is expressed in the units Gy Sv^-1^. The effective dose rate can be determined by applying the dose conversion factor from air kerma to effective dose, *C*_*E/K*_, for rotational geometry [17], and is also expressed in Sv Gy^-1^. Estimates of the effective dose rate to a population must also take into account the shielding effect of buildings and possibly snow, as well as the fraction of the day spent outdoors. Shielding by snow is taken into account by the factor *F*_*s*_ as a yearly average of the reduction in dose rate resulting from snow-covered ground compared to bare ground (corrected for the contribution from cosmic radiation), as calculated by Finck [12]. The shielding factor for buildings, *F*_b_, is defined as the dose rate inside the building divided by the dose rate outside the building, and a low value thus reflects a high shielding effect [12]. The outdoor occupancy factor, *F*_*out*_, used in this study was taken from Almgren et al. [18], i.e., 80% of the time is estimated to be spent indoors (1-*F*_*out*_). Combining the parameters described above gives the following equations, which can be used to predict the total effective dose rate from external exposure:

E˙(Cs134)=Aesd∙dCs∙fCs-134(t)∙r(t)∙hEmax*∙Fs∙CE/K∙[Fout+(1-Fout)∙Fb] (2)

E˙(Cs137)=Aesd∙dCs∙fCs-137(t)∙r(t)∙hEmax*∙Fs∙CE/K∙[Fout+(1-Fout)∙Fb] (3)

$$\dot{E}(short-lived)\;=\;A_{esd}∙d_{Cs}∙(1-f_{Cs-134}(t)-f_{Cs-137}(t))∙r(t)∙h_{Emax}^{*}∙F_{s}∙C_{E/K}∙[F_{out}+(1-F_{out})∙F_{b}]$$ (4)

The effective dose rate from the dominating internal sources ^134^Cs and ^137^Cs has been studied by Rääf et al. [8], [10], and applied to a population of Swedish hunter households by Tondel et al. [11]. The empirical relation between the regional-average ground deposition, *A*_*esd(county)*_, and effective internal dose rate for ^137^Cs is given by:

$$\dot{E}\;=\;\chi ∙A_{esd(county)}∙T_{ag(max)}∙[(1-e^{-\;\frac{ln2}{t_{1}}(t-t_{0})})∙c_{1}∙e^{-\;\frac{ln2}{t_{2}}(t-t_{0})}+c_{2}∙e^{-\;\frac{ln2}{t_{3}}(t-t_{0})}]∙k_{s}$$ (5)

where χ is a conversion coefficient between the body burden and the effective dose (mSv y^-1^ per (Bq kg^-1^)), and *A*_*esd(county)*_ is the county-averaged surface equivalent deposition of ^137^Cs (kBq m^-2^). Although it may be possible to find a relation between *A*_*esd*_ and *A*_*esd(county)*_, these parameters are treated as individual parameters to increase the versatility of the model. In a real situation, and using a detailed ^137^Cs deposition map, the population-based average deposition in a county will differ from the area-based county average, *A*_*esd(county)*_, depending on the range of fallout in that county, the effect of which will be addressed later in the sensitivity analysis. The parameter *T*_*ag(max)*_ (Bq kg^-1^ per kBq m^-2^) is a fitted parameter providing an approximate measure of the maximum transfer from local fallout on the ground, described as *A*_*esd(county)*_, to body burden. The terms *c*_*i*_, and *t*_*i*_ are fitted parameters that account for the time dependence of the ecological transfer pathways of radiocesium to the population. Time *t*_*0*_ is the time of the onset of fallout. The product $A_{esd(county)}∙T_{ag(max)}∙[(1-e^{-\;\frac{ln2}{t_{1}}(t-t_{0})})∙c_{1}∙e^{-\;\frac{ln2}{t_{2}}(t-t_{0})}+c_{2}∙e^{-\;\frac{ln2}{t_{3}}(t-t_{0})}]$ thus gives the whole-body activity concentration of ^137^Cs, *a*_*Cs*_, in Bq kg^-1^ at time *t* after the onset of fallout. The factor *k*_*s*_ accounts for the observed difference in Cs body concentration between men and women. The reference values of 0.61 for adult females and 1.00 for adult males are based on observations from the Swedish whole-body measurement database (described by Rääf et al. [8]), and have also been applied in a previous study of Swedish hunter households [11].

Relationships between effective dose rate and body burden have been calculated by Falk et al. [19], based on a computational model by Leggett et al. [20]. These relationships can be expressed by the conversion coefficient χ (mSv y^-1^/(Bq kg^-1^)) for ^134^Cs and ^137^Cs, respectively as:

χ(Cs134)=0.00164∙w0.188 (6)

and

χ(Cs137)=0.0014∙w0.111 (7)

Since the total Cs body burden, *a*_*Cs*_, is calculated from the surface equivalent deposition of ^137^Cs (Eq 5), it is necessary to account for the difference in physical half-life between the two Cs isotopes, as well as the isotopic ratio in the fallout. The correction factor for ^134^Cs can thus be written as $e^{\frac{ln2(2.06-30.2)}{2.06∙30.2}}∙FR$, where *FR* is the initial activity ratio of ^134^Cs/^137^Cs.

The equations predicting the total effective dose rate from external and internal exposure, assuming that the short-lived radionuclides only contribute to the long-term external dose rate, can then be written as follows (Eqs 8–10):

E˙(C134s)=Aesd⋅dCs⋅fCs−134(t)⋅r(t)⋅hEmax*⋅Fs⋅CE/K⋅[Fout+(1−Fout)⋅Fb]+0.00164⋅FR⋅eln2(2.06−30.2)2.06⋅30.2⋅Aesd(county)⋅Tag(max)⋅[(1−e−ln2t1(t−t0))⋅c1⋅e−ln2t2(t−t0)+c2⋅e−ln2t3(t−t0)]⋅w0.188⋅ks (8)

E˙(Cs137)=Aesd∙dCs∙fCs-137(t)∙r(t)∙hEmax*∙Fs∙CE/K∙[Fout+(1-Fout]∙Fb)+0.0014∙Aesd(county)∙Tag(max)∙[(1-e-ln2t1(t-t0))∙c1∙e-ln2t2(t-t0)+c2∙e-ln2t3(t-t0)]∙w0.111∙ks (9)

$$\dot{E}(short-lived)\;=\;A_{esd}∙d_{Cs}∙(1-f_{Cs-134}(t)-f_{Cs-137}(t))∙r(t)∙h_{Emax}^{*}∙F_{s}∙C_{E/K}∙[F_{out}+(1-F_{out})∙F_{b}]$$ (10)

Based on data from Rääf et al. [10], the parameters required in the equation describing the internal effective dose per unit ^137^Cs deposition (Eq 5) have been estimated for 4 sub-populations, in addition to hunters [11]. These sub-populations were rural non-farmers, farmers, urban residents and reindeer-herding Sami population (Table 1). The model allows these transfer factors to be adapted to the population under consideration when calculating the CED from the radiocesium contribution to the internal dose.

**Table 1:** Parameter values used to determine the internal effective dose for various sub-populations.

Sub-population	*T*_*ag(max)*_ (^137^Cs) [Bq kg^-1^/(kBq m^-2^)]	*t*_*1*_ [y]	*t*_*2*_ [y]	*t*_*3*_ [y]	*c*_*1*_ [–]	*c*_*2*_ [–]
Rural non-farmers	9.0	1.1	1.2	30	0.9	0.11
Farmers	11.0	1.0	1.0	15	0.9	0.10
Urban residents	11.0	1.0	0.75	15	1.0	0.10
Reindeer herders	200	2.0	2.0	15	1.0	0.10
Hunters	29.3	1.1	1.2	30	0.9	0.11

### Description of the scenarios

In this model, only generic parameters for adults and the effective dose to adults (age ≥15 y) are considered. The variation in external dose rate and in the cumulative effective dose over 50 years, *CED(50)*, were calculated for various scenarios related to mitigating activities. Three categories of scenarios are described: standard (S, OS, TH, TL), high-deposition (HD, HE, HR, HF) and low-deposition scenarios (LD, LE, LR, LF, V1), as defined in Table 2.

**Table 2:** Description of the modelled scenarios.

Scenario	Abbreviation	Description
Standard	S	Default parameter values in Table 3
Variation in outdoor occupation and shielding factor	OS	↑*F*_*out*_ ↑*F*_b_
High transfer	TH	↑*T*_*ag(max)*_
Low transfer	TL	↓*T*_*ag(max)*_
High deposition	HD	↑*A*_*esd*_ ↑*A*_*esd(county)*_
High deposition, maximum external component	HE	↑*A*_*esd*_ ↑ *A*_*esd(county)*_ ↑*FR* ↑*F*_*s*_ ↑*F*_*out*_ ↑*F*_b_
High deposition, relocation	HR	↑*A*_*esd*_ ↑*A*_*esd(county)*_; *F*_*out*_ = 0 and *F*_b_ = 0
High deposition, food restrictions	HF	↑*A*_*esd*_ ↑*A*_*esd(county)*_ ↓*T*_*ag(max)*_
Low deposition	LD	↓*A*_*esd*_ ↓*A*_*esd(county)*_
Low deposition, maximum external component	LE	↓*A*_*esd*_ ↓ *A*_*esd(county)*_ ↑*FR* ↑*F*_*s*_ ↑*F*_*out*_ ↑*F*_b_
Low deposition, relocation	LR	↓*A*_*esd*_ ↓ *A*_*esd(county)*_; *F*_*out*_ = 0 and *F*_b_ = 0
Low deposition, food restrictions	LF	↓*A*_*esd*_ ↓ *A*_*esd(county)*_ ↓*T*_*ag(max)*_
Highly variable deposition: high county-averaged deposition, low local deposition	V1	↓*A*_*esd*_ ↑*A*_*esd(county)*_

The high-deposition and low-deposition scenarios correspond to arbitrarily higher and lower areal activity concentrations, respectively, than that in the standard scenario (S). In the standard scenario, the parameters *A*_*esd*_ and *A*_*esd(county)*_ are both assumed to reflect a moderate or median areal activity concentration of 15 kBq m^-2^. This value corresponds to about ten times the global fallout from atmospheric nuclear weapon’s tests in the Northern hemisphere. The high-transfer (TH) and low-transfer (TL) scenarios are identical to the standard scenario but with two extreme values of transfer, *T*_*ag(max)*_, of 30.0 and 1.0 Bq kg^-1^/(kBq m^-2^), respectively. The OS scenario is another standard scenario with extreme outdoor occupancy (*F*_*out*_ = 1.0), which was compared with the HE and LE scenarios, which also have extreme outdoor occupancy (i.e. a high external component). The maximum contribution from the external exposure was modelled by setting the shielding factors for snow and buildings to 1 (meaning no shielding), and assuming that the population is constantly outdoors (i.e. *F*_*out*_ = 1.0). The values of *F*_*s*_ and *F*_b_ in the standard scenario were taken from Finck [12]; *F*_*out*_ from Almgren et al. [18], and *FR* from the isotopic ratio ^134^Cs/^137^Cs in the Chernobyl fallout over Sweden: 0.56 [13]. The values of the parameters used in each scenario are given in Table 3.

**Table 3:** Parameter values for adult males, used for calculations in the various scenarios listed in Table 2. (Shading indicates parameter values that are changed compared to the standard scenario (S)).

	Parameter											
Scenario	*A*_*esd*_ [kBq m^-2^]	*d*_*Cs*_ [mSv y^-1^per kBq m^-2^]	h*_Emax_ [Gy Sv^-1^]	*F*_*s*_ [–]	*C*_*E/K*_ [Gy Sv^-1^]	*F*_*out*_ [–]	*F*_b_ [–]	*A*_*esd(county)*_ [kBq m^-2^]	*w* [kg]	*k*_*s*_ [–]	*FR* [–]	*T*_*ag(max)*_ [–]
S	15	1.02	0.83	0.89	0.82	0.2	0.4	15	77.5	1	0.56	11.0
OS	15	1.02	0.83	0.89	0.82	1.0	1.0	15	77.5	1	0.56	11.0
TH	15	1.02	0.83	0.89	0.82	0.2	0.4	15	77.5	1	0.56	30.0
TL	15	1.02	0.83	0.89	0.82	0.2	0.4	15	77.5	1	0.56	1.0
HD	100	1.02	0.83	0.89	0.82	0.2	0.4	100	77.5	1	0.56	11.0
HE	100	1.02	0.83	1.0	0.82	1.0	1.0	100	77.5	1	1.0	11.0
HR	0	1.02	0.83	0.89	0.82	0.0	0.0	100	77.5	1	0.56	11.0
HF	100	1.02	0.83	0.89	0.82	0.2	0.4	100	77.5	1	0.56	1.0
LD	5	1.02	0.83	0.89	0.82	0.2	0.4	5	77.5	1	0.56	11.0
LE	5	1.02	0.83	1.0	0.82	1.0	1.0	5	77.5	1	1.0	11.0
LR	0	1.02	0.83	0.89	0.82	0.0	0.0	5	77.5	1	0.56	11.0
LF	5	1.02	0.83	0.89	0.82	0.2	0.4	5	77.5	1	0.56	1.0
V1	5	1.02	0.83	0.89	0.82	0.2	0.4	100	77.5	1	0.56	11.0

Permanent relocation (HR, LR) was parameterized by completely removing the external component (*F*_*out*_ = 0 and *F*_b_ = 0), but can also be achieved by setting *A*_*esd*_ = 0. However, since this action does not necessarily mean that no contaminated food is consumed, the internal exposure is still modelled using different assumptions regarding *T*_*ag(max)*_. The internal dose was modelled from low transfer in a temperate zone of 1.0 to a maximum transfer with a *T*_*ag(max)*_ value of 29.3 representing Swedish hunters. The lower value of *T*_*ag(max)*_ represents the situation reported from Japan, where a relatively low correlation between ground deposition and whole-body contents of ^137^Cs in local residents was found after the Fukushima Daiichi accident [21]. A scenario in which the areal activity concentration within a region was highly variable was also modelled (V1). This may be the case in a real fallout scenario where large local variations in deposition density are not reflected in the regional average *A*_*esd(county)*_, e.g. deposition in a rural food-producing area with a low population density. In this case, a high value was assigned to the regional average value of ^137^Cs deposition, (*A*_*esd(county)*_ = 100 kBq m^-2^), while the local deposition at the site of the dwelling, *A*_*esd*_, was assigned a low value of 5 kBq m^-2^.

**Fig 1:**
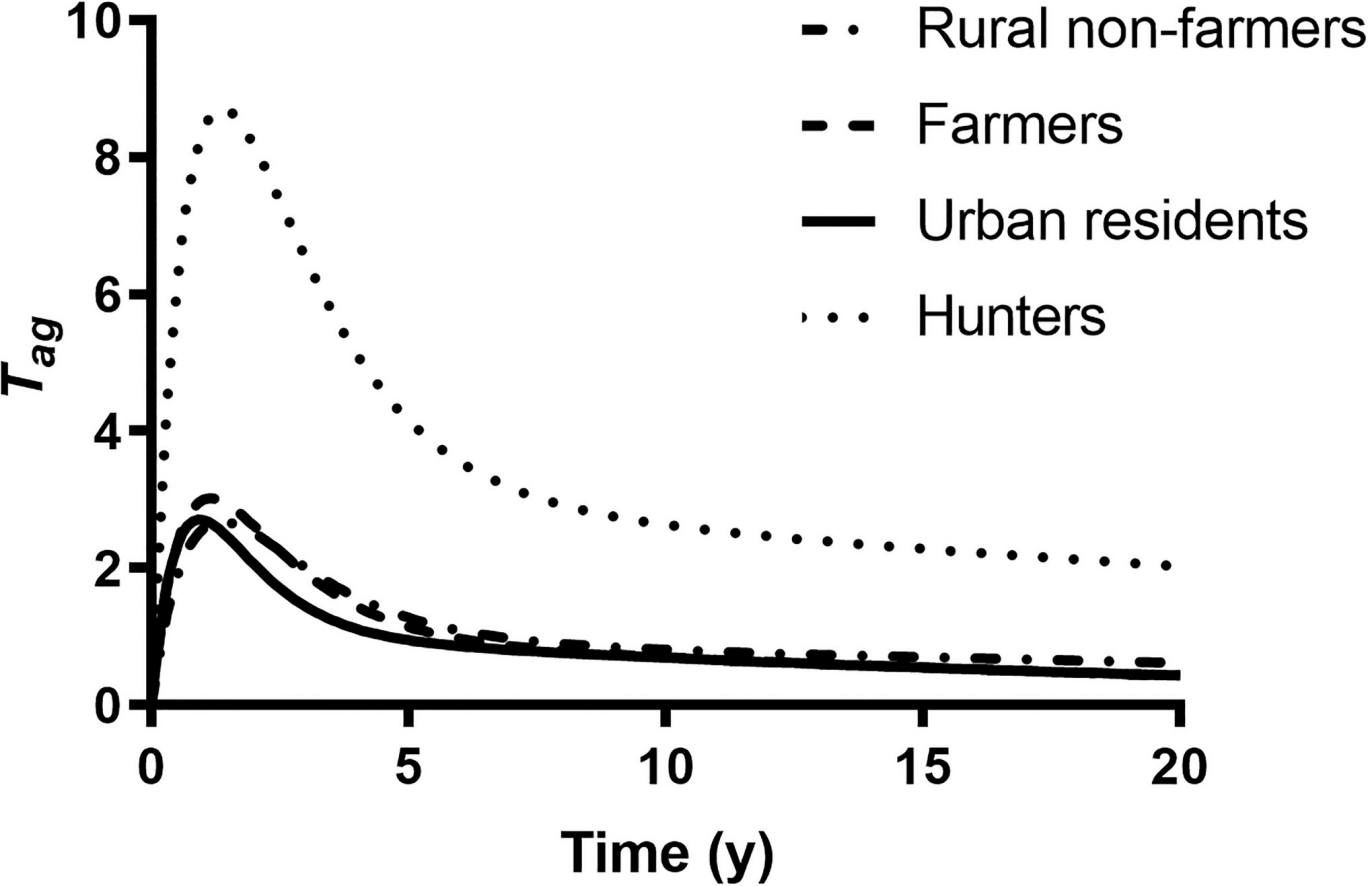
Time-dependent transfer of ^137^Cs to various sub-populations.

The parameters for *T*_*ag*_(*t*) for an urban resident were chosen to simulate the internal exposure pathway (Table 1) since these can be assumed to represent the average urban population, according to Fig 1, where *T*_*ag*_(*t*) is shown for 4 of the sub-populations. As mentioned above, the value of *T*_*ag(max)*_ for an urban population also agrees reasonably with international observations of time-integrated radioecological transfer made in the 1960s and 1970s for nuclear weapons fallout [10]. The radioecological transfer to the Swedish urban population probably reflects highly regulated food consumption, whereas the transfer to hunters does not (Table 1). Observations after the Fukushima Daiichi accident in 2011 (see e.g. [21]) showed that very strict food regulations can reduce *T*_*ag*_ to a value considerably lower than that for the Swedish urban population, and this value was therefore reduced to 1.0 kg^-1^ per kBq m^-2^ in the scenarios involving very strict food restrictions (HF, LF and TL, Table 2).

The body weight was chosen to reflect an average adult Swedish male, 77.5 kg [22]. The average body weight of adult Swedish women is 63.3 kg, and the difference in effective dose between men and women will thus be negligible in these calculations ((77.5/63.3)^-0.188^ = 0.96 for ^134^Cs and (77.5/63.3)^-0.111^ = 0.98 for ^137^Cs)). Since all calculations were performed for adult males, the internal effective dose to women can be found by multiplying the internal dose by *k*_*s*_ = 0.61. However, the external dose estimates are identical for both sexes.

The cumulated effective dose to children was considered in the calculation of the total lifetime effective dose up to 84 years of age (the average life expectancy for females in Sweden [23]), *E*_*84*_, for individuals of various ages at the time of the fallout. In all scenario calculations, individuals were regarded as children up to and including 15 years of age, and the age-dependent parameters *w* and *C*_*E/K*_, for rotational geometry were set to 35 kg [24] and 0.89 Sv Gy^-1^ [25], respectively.

### Sensitivity analysis

Several of the parameters used in the model (Eqs 8–10) are associated with large uncertainties and high variability. To determine which parameters in the model have the greatest effect on the final *CED(50)* estimates, sensitivity analyses were performed by: (*i*) varying one parameter at a time by ±50% of the central estimate while the remaining parameters retained the values used in the standard scenario (S) and the high-transfer scenario (TH), respectively; and (*ii*) by performing Monte Carlo simulations, where all the parameters were sampled from probability density functions and varied simultaneously (Table 4). When possible, the probability density function associated with each parameter was based on previous observations. For example, it was found that the intra-variability in the ^137^Cs uptake in human populations was described by a lognormal distribution [8]. The variability in local deposition density (*A*_*esd*_) and county average *A*_esd(*county*)_ was modelled as lognormal variables where the sampled *A*_esd(*county*)_ in each run was used as the expectation value for the lognormal distribution of *A*_*esd*_.

**Table 4:** Parameter values and probability distributions used in the sensitivity analysis. Central estimates of parameters were those used in the standard scenario (S). The references for the choice of the minimum and maximum parameter values are given in the footnotes. Gray shading indicates parameters that are not applicable for the respective type of distribution.

Parameter	Prob. density function	Mean	SD	Central estimate	Min.	Max.	Note
***A***_***esd***_ [kBq m^-2^]	Lognormal	0.9793·*A*_*esd*(*county)*_	1.252				1
***d***_***Cs***_ [mSv y^-1^per kBq m^-2^]	Normal	1.02	0.67				2
***f(t)*** [–]	Normal	*f(τ)*	0.2· *f(τ)*				3
***r(t)*** [–]	Normal	*r(τ)*	0.2· *r(τ)*			1.0	4
h*_Emax_ [Gy Sv^-1^]	Uniform			0.80	0.76	0.85	5
***F***_***s***_ [–]	Uniform			0.89	0.81	0.97	6
***C***_***E/K***_ [Sv Gy^-1^]	Uniform			0.80	0.70	0.90	7
***F***_***out***_ [–]	Triangular			0.2	0.1	0.3	8
***F***_**b**_ [–]	Triangular			0.4	0.25	0.55	9
***A***_***esd(county)***_ [kBq m^-2^]	Lognormal	Variable	20%				10
***W*** [kg]	Normal	80	10				11
***k***_***s***_ [–]	Uniform			0.8	0.6	1.0	12
***FR*** [–]	Uniform			0.6	0.2	1.0	13
***T***_***ag(max)***_ [–]	Lognormal	11.0	11.0				14
***t***_***1***_ [y]	Normal	1.0	5%				14
***t***_***2***_ [y]	Normal	0.75	5%				14
***t***_***3***_ [y]	Norm	15	5%				14
***c***_***1***_ [–]	Norm	1.0	5%				14
***c***_***2***_ [–]	Norm	0.10	5%				14

^1^Geometric mean and standard deviation. Based on data from measurements by the Environmental Health Departments at Swedish municipalities.

^2^Data from Jönsson et al. [4]. The standard deviation also accounts for variations in the ratio between ambient dose equivalent rate and *A*_*esd*_.

^3^The value of the function *f*(*t*) at each time step *τ* is used as input. Data from Jönsson et al. [4].

^4^The value of the function *r*(*t*) at each time step *τ* is used as input. The probability density function is truncated at 1.0. Data from Jönsson et al. [4].

^5^Data from International Commission on Radiation Units and Measurements [16] for photon energies between 300 and 1000 keV.

^6^Data from Finck [12].

^7^Data from International Commission on Radiological Protection [17] for photon energies between 150 and 1000 keV, for rotational and isotropic exposure geometry.

^8^Data from Almgren et al. [18].

^9^Data from Finck [12].

^10^Parameter value chosen based on simulations.

^11^Estimate based on data from Samuelsson and Hagman [22]: 77.5 kg for adult men (>18 years).

^12^Data from Tondel et al. [11].

^13^Central estimate from Edvarson [13].

^14^Data from Rääf et al. [10] and Tondel et al. [11].

Monte Carlo simulations were performed with parameter values sampled from the probability density functions given in Table 4, using the software @RISK 7.5 (Palisade Corporation, UK). The value of *A*_*esd(county)*_ used was 15 kBq m^-2^. Each probability density function was sampled using Latin hypercube sampling, and each simulation was run for 5000 iterations. As in the simple sensitivity analysis where one parameter was varied at a time, the CED for adult males during 50 y was chosen as output. In this case, *CED(50)* is given as a distribution of 5000 outputs, each generated from a random choice of parameter values based on the probability density function assumed for each parameter. Parameter distributions were truncated to return only positive values.

### Statistical analysis

The model was implemented in Microsoft Excel 2016 (Microsoft Corporation, USA). The sensitivity analysis utilizing Monte Carlo simulations was performed using @RISK 7.5.

## Results and discussion

### Scenario modelling

**Fig 2:**
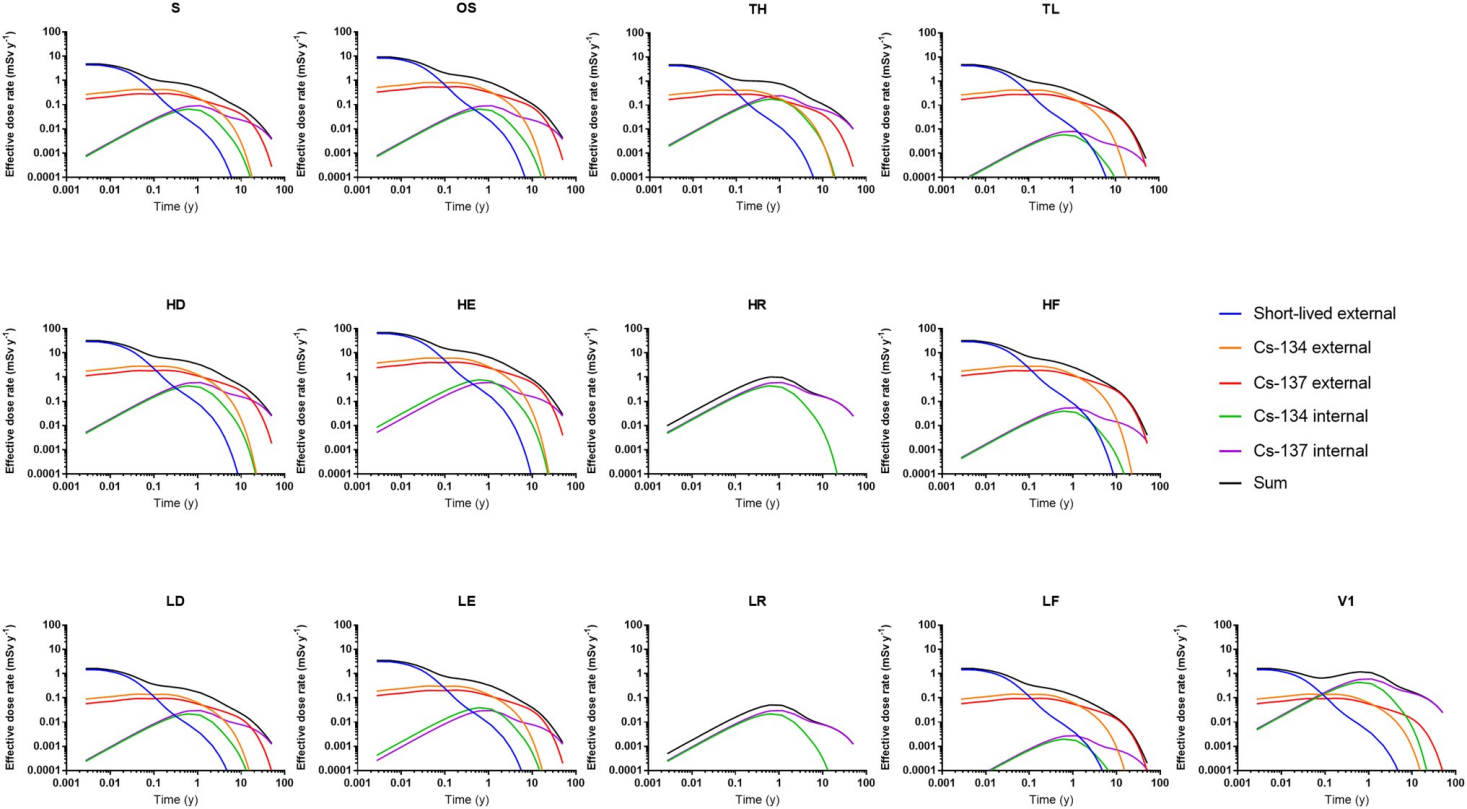
Effective dose rate (mSv y^-1^) for adult males during the 50 years following fallout, assuming the various scenarios described in Table 2.

The time dependence of the effective dose rate (mSv y^-1^) for the various scenarios is shown in Fig 2. The appearance of the curves for the standard scenario and the high-/low-deposition scenarios (S, HD & LD) is, as expected, similar, but with differences in magnitude reflecting the different equivalent surface deposition, *A*_*esd*_.

For all scenarios except those involving relocation (HR, LR) and food restrictions (HF, LF), the total effective dose rate was found to be dominated by the internal and external contribution from ^137^Cs 10 years after the accident, and the value had decreased by about three orders of magnitude 50 years after the accident. Maximizing the external component (HE & LE) increased the total effective dose considerably, and this dominated completely over the internal contribution from ^137^Cs.

**Fig 3:**
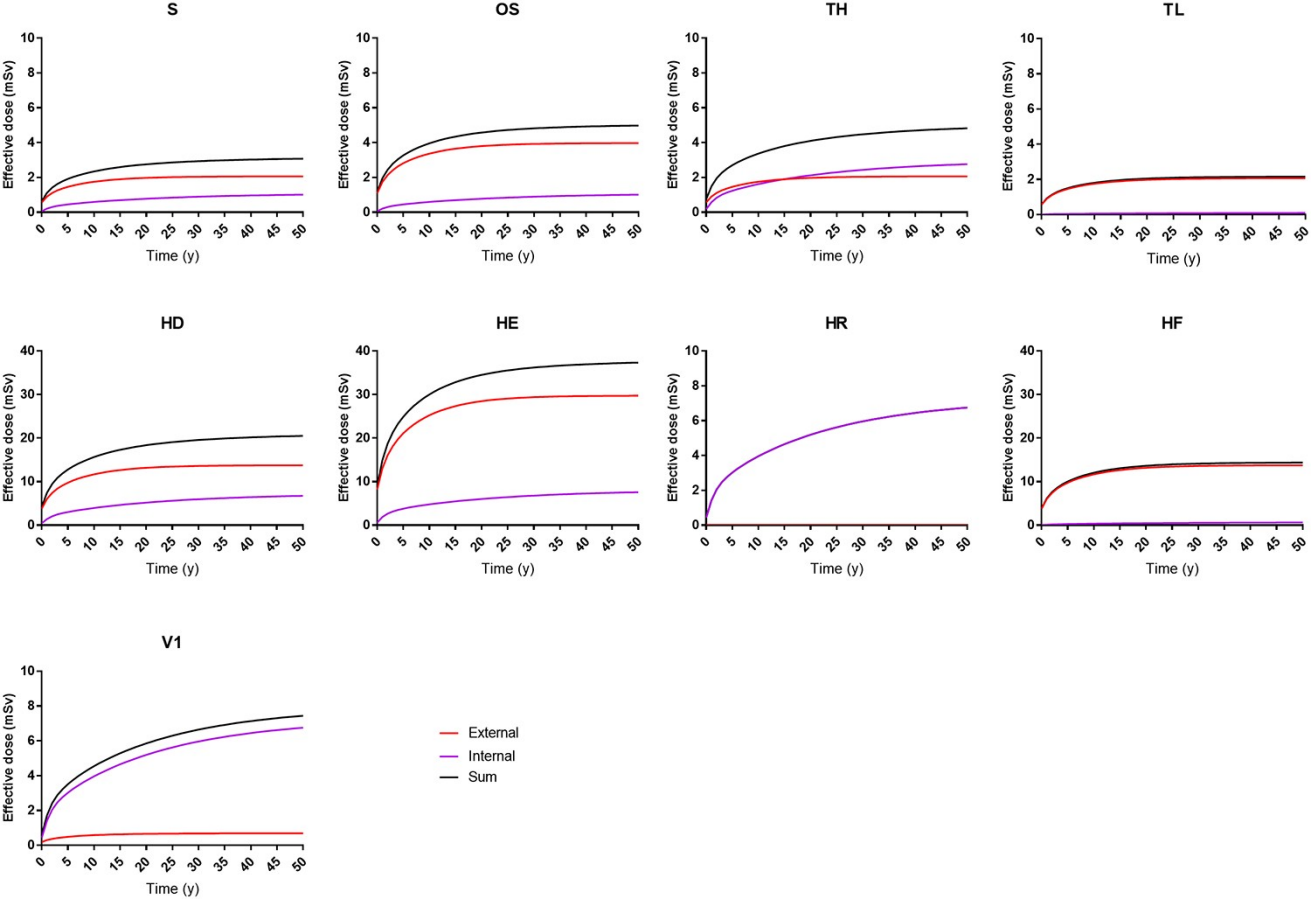
Cumulative effective dose (mSv) for adult males during 50 years, assuming the various scenarios described in Table 2. Note the varying scales on the dose axis.

The CED as a function of time after fallout for the key scenarios is presented in Fig 3 for the same period. The CED curves were obtained by integrating the dose rates shown in Fig 2, where the contributions from external and internal exposures have been summed separately. The figure thus shows the contributions to the total CED from external (short-lived radionuclides, ^134^Cs and ^137^Cs) and internal exposure (^134^Cs and ^137^Cs). Interestingly, the cumulative dose has not reached a plateau after 50 years in the high-deposition scenarios. The time at which a plateau is reached, *t*_95_, expressed as 95% of the maximum CED, *CED*_max_, for the various scenarios, is given in Table 5. It can also be seen that the only scenario in which the curve describing the internal component crosses the curve describing the external component is when a high transfer is assumed in the standard scenario (TH). This occurs about 15 years after the fallout (Fig 3).

**Table 5:** Cumulative effective dose (mSv) for adult males, *CED(50)*, *CED(50)* per unit deposited activity, time (*t*_95_) at which a plateau is reached (95%), and maximum CED, *CED*_*max*_, assuming the various scenarios described in Table 2.

Scenario	External (mSv)	Internal (mSv)	*CED(50)* (mSv)	*CED(50)*/*A*_esd_ (mSv per kBq m^-2^)	*t*_95_ (y)	*CED*_*max*_ (mSv)
S	2.06	1.01	3.08	0.205	35	3.14
HD	13.8	6.75	20.5	0.205	35	21.0
HE	29.7	7.59	37.3	0.373	27	37.8
HR	0	6.75	6.75	0.675	53	7.19
HF	13.8	0.61	14.4	0.144	21	14.4
V1	0.69	6.75	7.44	1.488	52	7.87
OS	3.97	1.01	4.98	0.332	29	5.05
TH	2.06	2.76	4.83	0.322	44	5.01
TL	2.06	0.092	2.16	0.144	21	2.16

**Fig 4:**
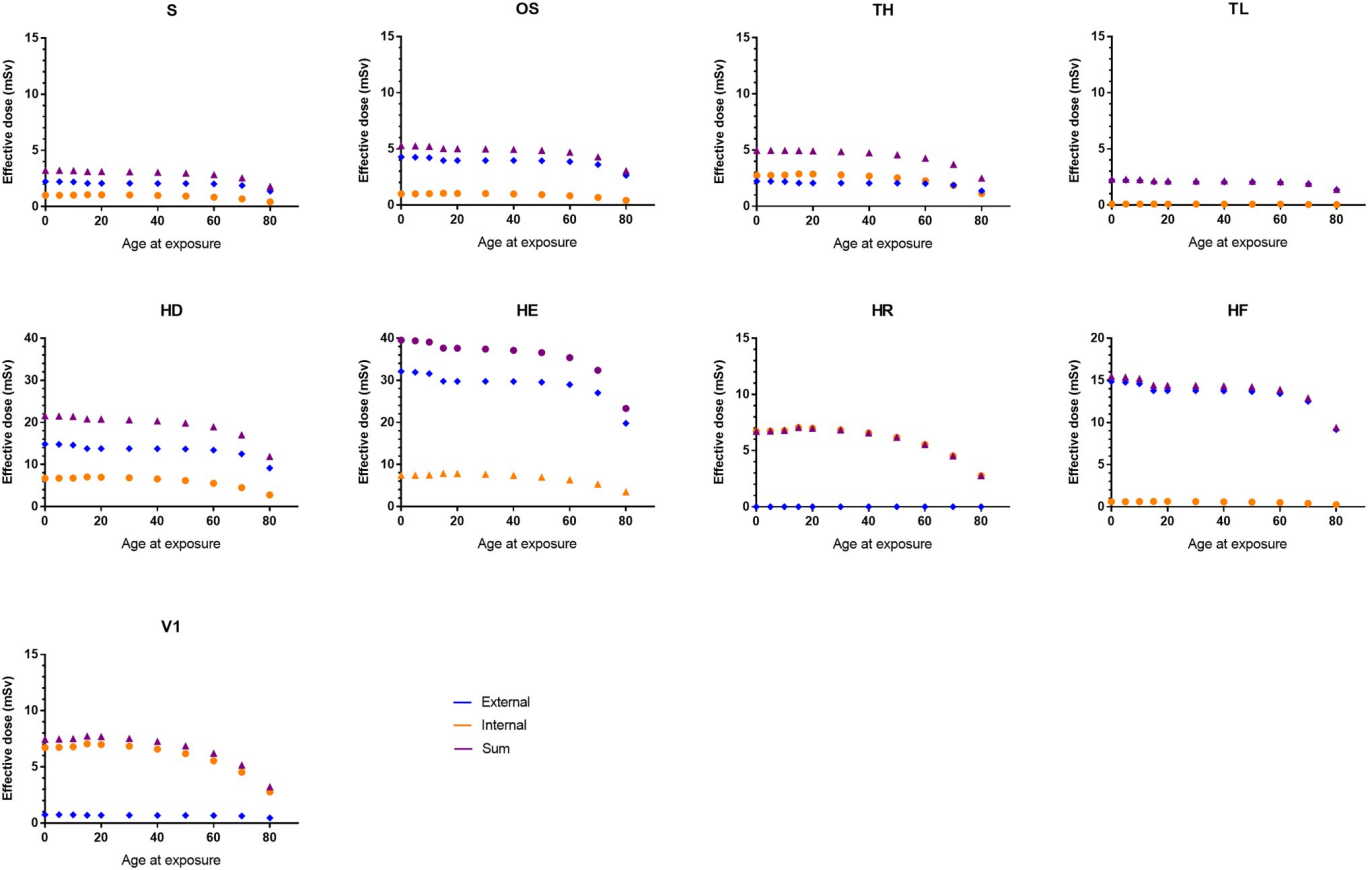
Lifetime effective dose (mSv) up to age 84, E84, for males under various scenarios, as a function of age at the onset of exposure. Note the different scales for three of the high-deposition scenarios.

The total effective dose up to age 84 y, *E*_*84*_, depending on the age of exposure, is shown in Fig 4. It should be borne in mind that the transient contributions from inhalation and cloud shine have not been considered here, even in the scenarios where long-term mitigating actions are not carried out. Based on data from, for example, Finck [12], the effective dose contributions from inhalation and cloud shine in the areas in Sweden most affected by the Chernobyl NPP accident, with a value of *A*_*esd*_ of about 50 kBq m^-2^, were estimated to be less than 20 μSv. Although more recent estimates have revealed that the maximum inhalation doses in Sweden were on the island of Gotland, where levels up to 0.2 mSv may have been reached [26], this is still considered negligible compared to the *CED(50)* values estimated in the present study. The corresponding contribution from the transfer of ^131^I through dairy milk is also considered to be small, being about 0.01 mSv/(kBq m^-2^) per unit equivalent surface deposition of ^137^Cs, based on data from a detailed study of three dairy farms in Sweden in 1986 [27]. These two examples thus illustrate the negligible impact these initial exposure pathways may have in relation to the long-term pathways, provided mitigating actions are instigated during the early phase of the accident.

**Fig 5:**
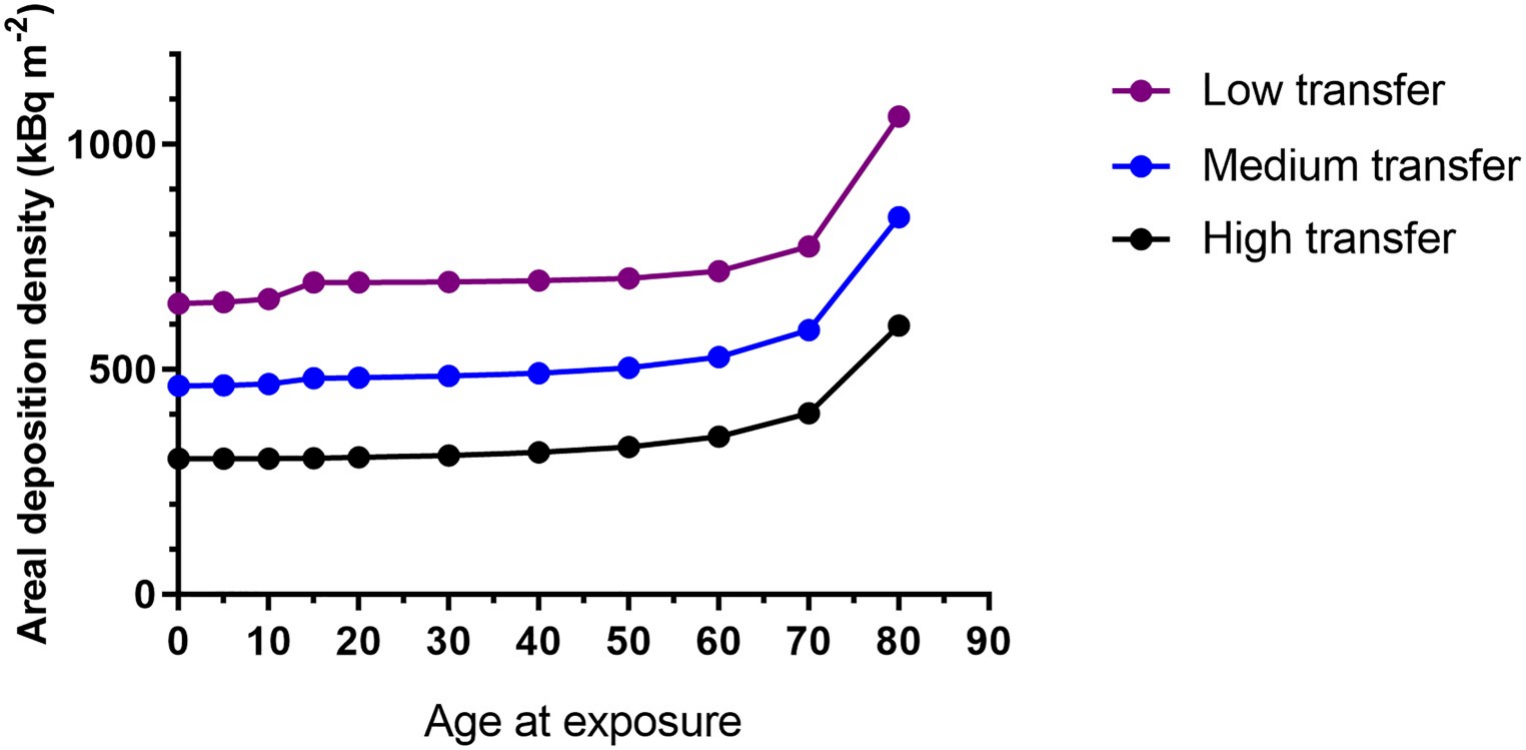
Areal deposition density of ^137^Cs that would result in a cumulative effective dose of 100 mSv up to an age of 84 years as a function of age at the onset of exposure, assuming low-, medium- and high-transfer scenarios, corresponding to *T*_*ag(max)*_ of 1.0, 11.0 and 30.0, respectively.

The calculations of cumulative effective dose (mSv) up to age 84 y, *E*_*84*_, were used to estimate the areal deposition density that would result in a lifetime effective dose of 100 mSv, depending on the age at fallout (Fig 5). These calculations were performed using the fixed parameter values from the standard scenario (S), and varying *T*_*ag(max)*_ to represent three scenarios: low- (TL), medium- (S) and high-transfer (TH), and the values chosen for *T*_*ag(max)*_ from Table 3 were thus 1.0, 11.0 and 30.0, respectively.

### Sensitivity analysis

**Fig 6:**
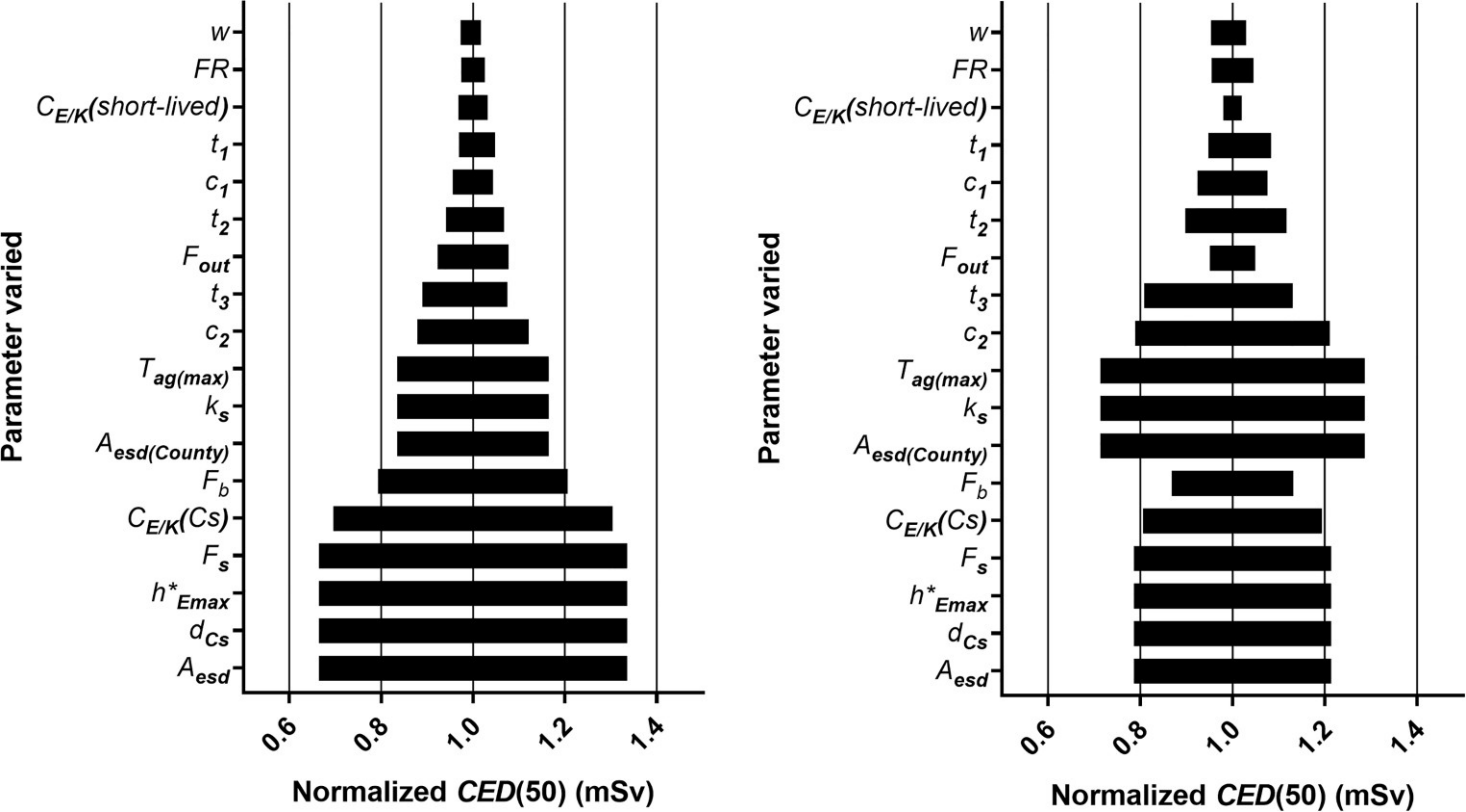
Results of the sensitivity analysis of the cumulative effective dose for adult males after 50 years, *CED(50)*. Each parameter was varied by ±50% while all the other parameters retained the values they had in the standard scenario (left), or in the high-transfer scenario (right).

The results of the simple sensitivity analysis are shown in Fig 6. The time-dependent parameters *r*(t) and *f*(t) were excluded from this analysis. It can be seen from the left part of Fig 6 (scenario S) that the parameters determining the time dependence of the transfer of ^134^Cs and ^137^Cs whole-body concentrations (*c*_i_, *t*_i_) have a limited impact on *CED(50)*. *T*_*ag(max)*_ also has a minor effect on *CED(50)*. The relative importance of these parameters increases in the high-transfer scenario (scenario TH), due to the higher contribution from the internal dose (Fig 6, right part). The body weight (*w*), conversion factor between air kerma rate and effective dose rate, *C*_*E/K*_, for short-lived isotopes, the ^134^Cs/^137^Cs isotopic ratio (*FR*), occupancy factor (*F*_out_), correction factor male/female (*k*_s_), and county-averaged Cs deposition (*A*_esd*(county)*_) all have a relatively small effect on *CED(50)* in the Standard scenario, in comparison to local *A*_*esd*_. However, since both *k*_s_ and *A*_esd*(county)*_ are linked to the transfer of cesium from deposition to the body, the contribution from both of these parameters is increased in the high-transfer scenario. In the standard scenario, the parameters with the greatest influence on *CED(50)* are the shielding factors (for buildings and snow), and the three coefficients *C*_E/K_, h*_Emax_ and *d*_*Cs*_. These parameters are of relatively less importance in the high-transfer scenario where instead parameters linked to internal dose dominate. However, the ^137^Cs activity at the location of the dwelling (*A*_*esd*_) is most important for the 50-year dose in the standard scenario (S). As already seen in Fig 3, the external component dominates *CED(50)*, and the parameters determining this component thus have the greatest impact. Some parameters determining the external exposure also influenced *CED(50)* in the high-transfer scenario, which is consistent with the results shown in Fig 3, where the external and internal contributions converge about 15 y after fallout.

**Fig 7:**
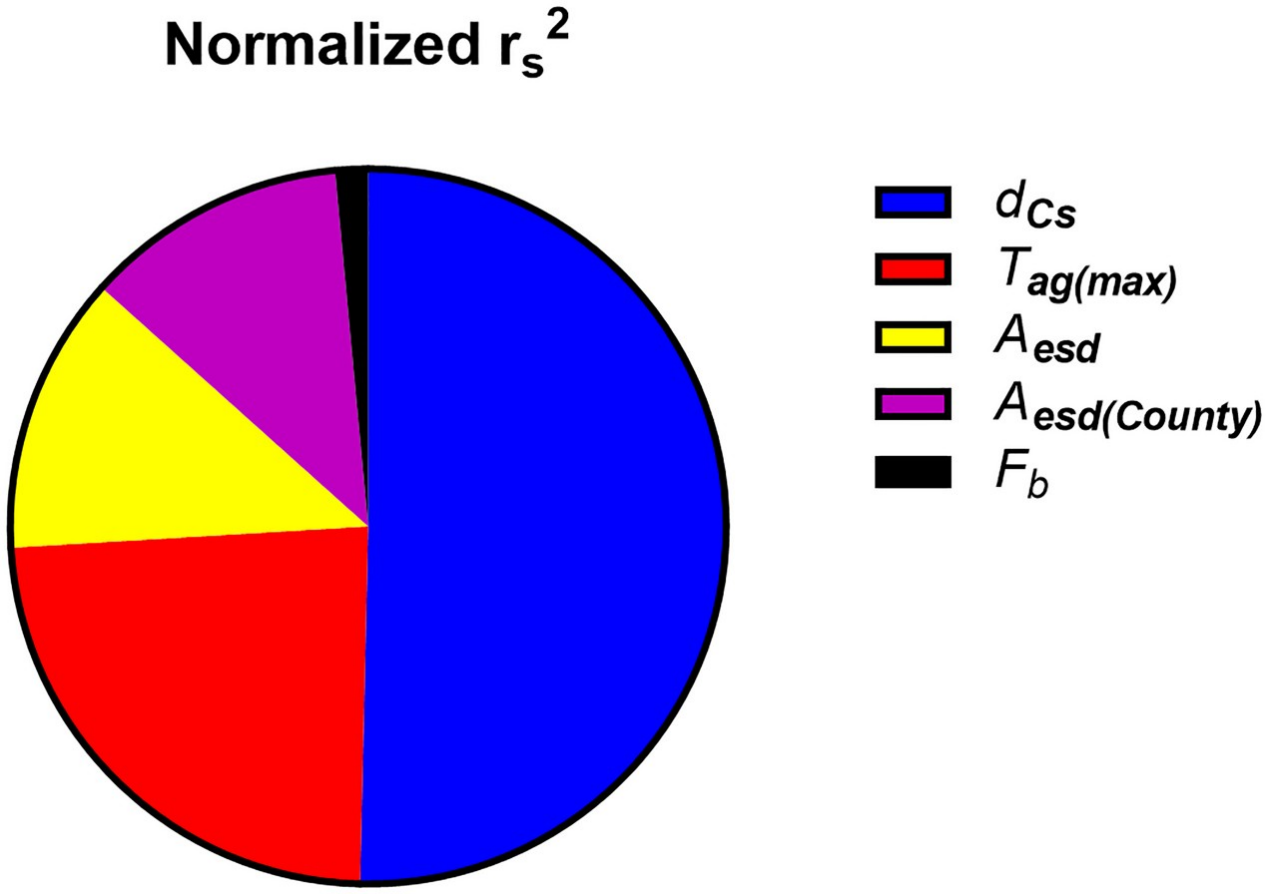
Fraction of correlation contributed by each parameter given in Table 6.

**Fig 8:**
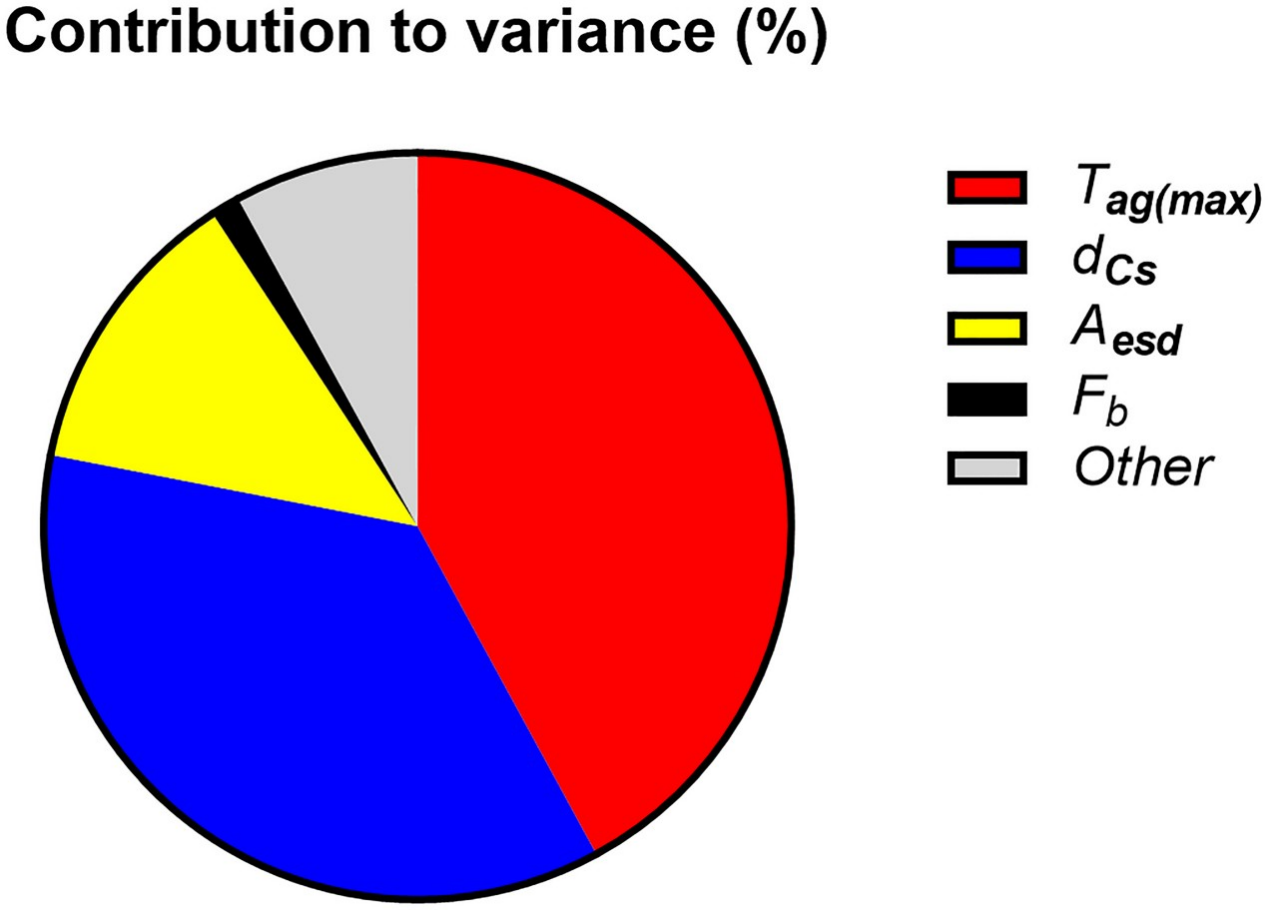
Contribution to the total variance for parameters accounting for more than 1% of the variance in *CED(50)*. The segment “Other” includes all other parameters; the greatest contributions being from *k*_*s*_, *C*_*E/K*_, *F*_*s*_, *F*_*out*_, *A*_*esd(county)*_, *FR*, h*_Emax_, *r(t)* and *c*_*2*_.

The parameters that had the greatest effects on *CED(50)* in the standard scenario, according to the sensitivity analysis performed with Monte Carlo simulations, are given in Table 6, together with the values of the Spearman rank correlation coefficient, *r*_*s*_. The Spearman correlation coefficient is a measure of the degree of association between two variables, but in contrast to the Pearson correlation coefficient, the variables do not need to have a linear relationship. Thus, the influence of very large or very small parameter values are mitigated since the correlation is determined by the rank numbers of pairwise parameter values in each iteration, instead of the actual parameter values. Since correlation can be either positive or negative, Fig 7 shows the fraction of the correlation contributed by each parameter in Table 6, given as the normalized value of *r*_*s*_^2^, for comparison. The contribution to the variance in *CED*(50) was calculated using @RISK, and is shown in Fig 8 for the parameters that contributed more than 1%. Since each parameter value is randomly chosen in each iteration, and will thus differ to some extent, each parameter value in an iteration is grouped by the software into bins of equal size. The change in output, in this case *CED*(50) associated with each bin is determined and used in the ranking of the parameters.

**Table 6:** Spearman rank correlation coefficient, *r*_*s*_, for *CED(50)*. Only parameters with a correlation coefficient greater than 0.1 are given.

Parameter	*r*_*s*_
*d*_*Cs*_	0.70
*T*_*ag(max)*_	0.48
*A*_*esd*_	0.35
*A*_*esd(county)*_	0.34
*F*_*b*_	0.12

It can be seen from Table 6 and Fig 7 that *d*_Cs_ is strongly correlated with *CED(50)*. However, as can be seen from Fig 8, the parameter with the strongest correlation does not necessarily have the greatest influence on the final result. The contribution to the variance is also related to the variability and the average of that parameter.

**Fig 9:**
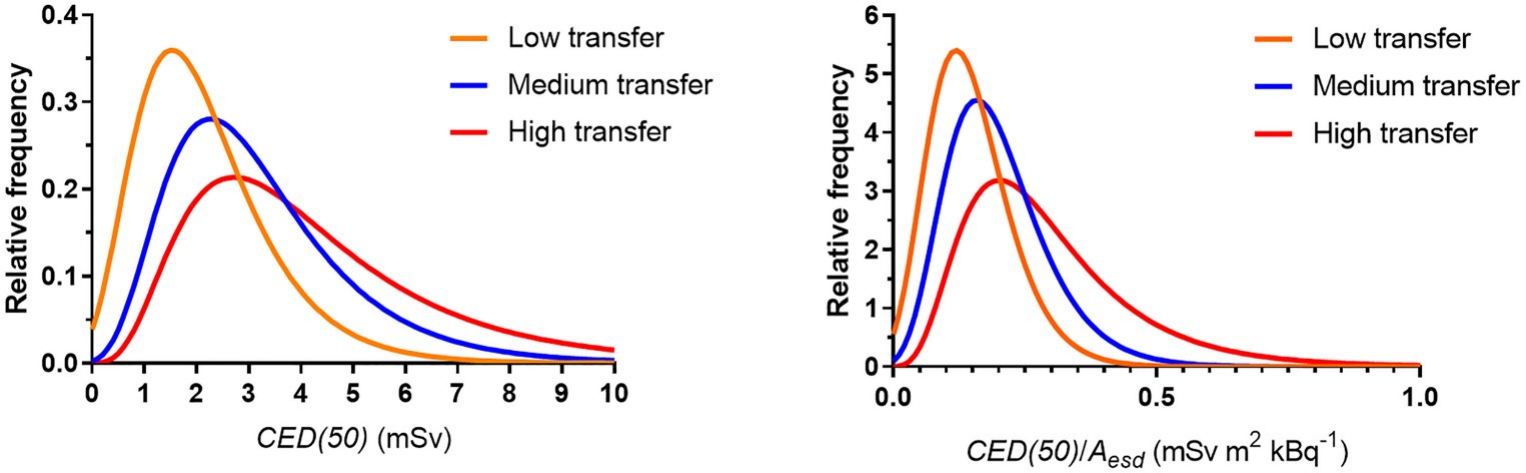
Distribution of *CED(50)* (left) and *CED(50)*/*A*_esd_ (right) for adult males, fitted by lognormal distributions. The curves representing medium transfer (standard scenario) were calculated by Monte Carlo simulation with *T*_*ag(max)*_ = 11, while the low-transfer and high-transfer curves were calculated using *T*_*ag(max)*_ = 1 and *T*_*ag(max)*_ = 30, respectively.

The distribution of *CED(50)* for adult males for varying transfer is shown in Fig 9 (left part of the figure). In these simulations the standard deviation in the lognormal distribution for *A*_*esd(county)*_ was set equal to the mean value in order to reflect a large uncertainty in the deposition density. The information in the figure provides a basis for estimates of *CED(50)* from early (and uncertain) measurements of the deposition density shortly after a fallout event. The right part of Fig 9 shows the distribution of *CED(50)*/*A*_esd_.

### Validation

**Fig 10:**
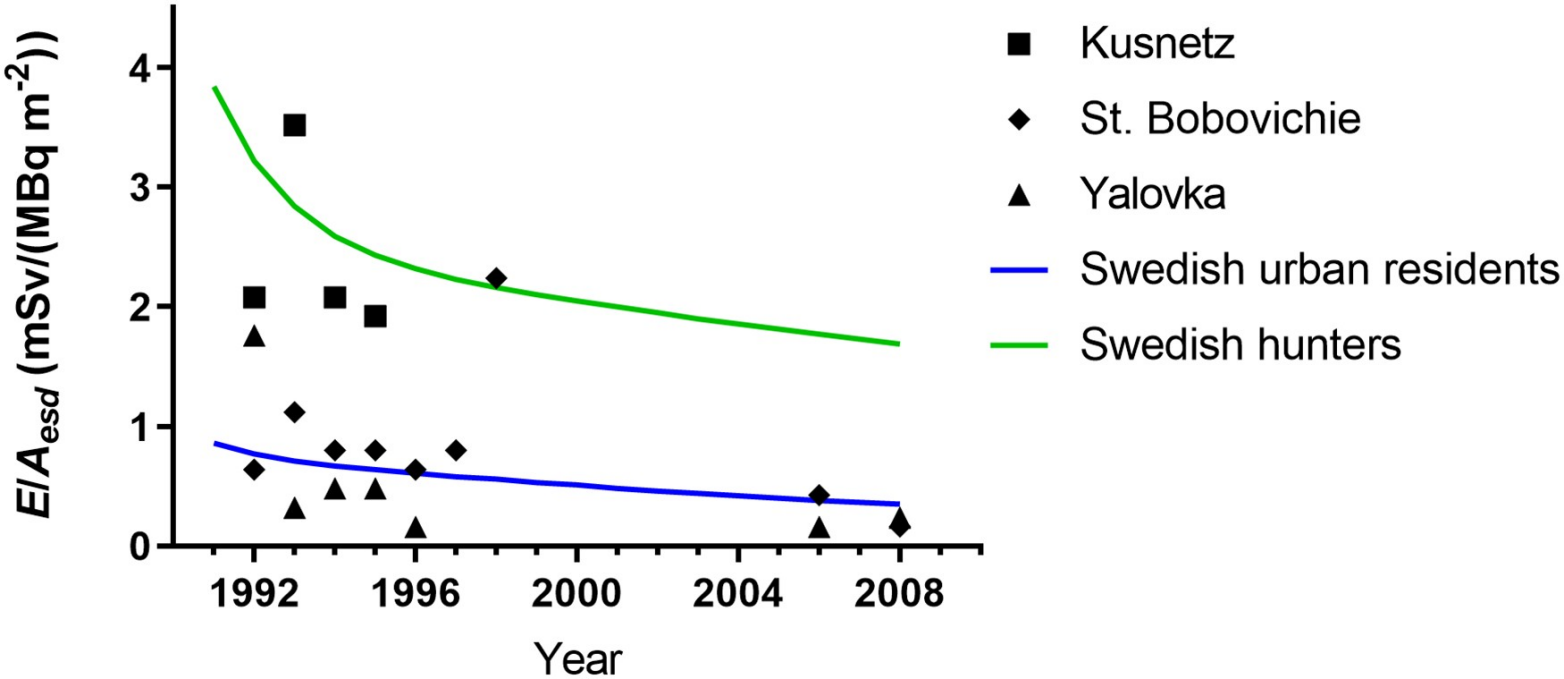
Yearly effective dose, *E*, per unit areal activity deposition of ^137^Cs, *A*_*esd*_, calculated with the present model for urban residents and hunters, together with estimates based on measurements performed in three Russian villages [28], [29].

The predictions by the model were compared to estimates of the effective dose to inhabitants in Russia (western Bryansk region) based on *in vivo* measurements and measurements of external dose rates in three Russian villages: Kusnetz, St. Bobovichie and Yalovka [28], [29]. Fig 10 shows the model predictions of yearly effective dose per unit ^137^Cs ground activity deposition for urban residents and hunters, using parameter values from Table 1, together with the estimates reported by Thornberg et al. and Bernhardsson et al. [28], [29]. It can be seen that the model predictions agree quite well; data from Kusnetz agrees with the predictions obtained using model parameters for Swedish hunters, whereas the yearly effective dose in the other two villages agree with the corresponding prediction for Swedish urban populations, indicating that the model is able to make predictions for other geographical areas.

## Conclusions

To the best of our knowledge, this study is the first attempt to develop a model in which both the external and internal exposure pathways are implicitly linked to a fallout map of ^137^Cs after a NPP accident. Our model also shows that the long-term total effective dose, expressed as *CED(50)*, including both internal and external dose contributions, per unit ^137^Cs deposition, ranges from 0.14 mSv/kBq m^-2^ (scenarios with food restrictions and low transfer, respectively) to 1.5 mSv/kBq m^-2^ (scenario with highly variable deposition). This is consistent with estimates of the external cumulative exposure in terms of air kerma over 30 years, which have been reported to be 970 and 570 mGy for an initial areal ^137^Cs deposition of 1 MBq m^-2^ following the Chernobyl and Fukushima Daiichi NPP accidents, respectively [30]. Given a scenario with a transfer corresponding to standard Scandinavian conditions, our model predicts that an initial areal ^137^Cs deposition of about 450 kBq/m^-2^ will result in a lifetime effective dose exceeding 100 mSv.

The most important parameters governing *CED(50)* for various adult populations appear to be the correlation factor between the local areal deposition of ^137^Cs and the maximum initial ambient dose rate, *d*_*Cs*_; the maximum transfer factor, *T*_*ag(max)*_; the local areal deposition of ^137^Cs, *A*_*esd*_; and the region-averaged ^137^Cs deposition, *A*_*esd(county)*_. It is therefore important for the prediction of *CED*(50) in a future fallout scenario involving releases similar to those from the Chernobyl or Fukushima Daiichi NPP accidents, that mapping of the local *A*_*esd*_ is carried out immediately, and that accurate transfer factors for the populations affected are identified in the literature based on previous studies. The model proposed here can also be used for the prediction of lifetime doses under different assumptions, provided *A*_*esd*_ is defined in the region.

## Supporting information

S1 Dataset Data in table format corresponding to all figures can be found in the file S1 Dataset. (XLSX)
